# Decoding the interconnected splicing patterns of hepatitis B virus and host using large language and deep learning models

**DOI:** 10.1099/mgen.0.001616

**Published:** 2026-01-16

**Authors:** Chun Shen Lim, Chris M. Brown

**Affiliations:** 1 Department of Biochemistry, Faculty of Biomedical Sciences, University of Otago, Dunedin, New Zealand; 2 Genetics Otago, University of Otago, Dunedin, New Zealand; 3 Maurice Wilkins Centre for Molecular Biodiscovery, Centres of Research Excellence, Auckland, New Zealand

**Keywords:** artificial intelligence, deep sequencing, large language model, splicing efficiency, virus–host coevolution

## Abstract

Hepatitis B virus (HBV) infection causes one million deaths annually and remains a major driver of hepatocellular carcinoma. Despite its compact 3.2 kb genome, HBV exhibits extensive alternative splicing. HBV splice variants contribute to immune evasion and reduce the likelihood of achieving a functional cure. Here, we show that HBV splicing efficiency – quantified from 279 RNA-sequencing libraries of HBV-associated liver biopsies and cultured cells – correlates more strongly with disease progression than the overall proportion of spliced HBV RNA, the latter of which has been proposed as an emerging biomarker. All HBV splice sites are embedded within protein-coding regions, forming a gene structure distinct from typical host splice sites. To decode the sequence determinants of HBV splicing, we apply SpliceBERT and OpenSpliceAI to 4,706 HBV genomes. These models reveal that HBV splice donor sites share features with host splice donor sites, whereas HBV splice acceptor sites are more cryptic. These patterns likely reflect constraints imposed by HBV’s compact genome, which must accommodate overlapping protein-coding regions. Motif conservation and splicing propensity analyses across HBV genomes reveal context- and genotype-specific splicing patterns, indicating regulation by sequence context. HBV genotypes may have coevolved with their human hosts to exploit suboptimal but spliceable host-like motifs without disrupting their gene structure, supporting mechanisms of viral persistence and immune evasion. This study demonstrates the utility of artificial intelligence in decoding viral splicing patterns and provides a framework for investigating co-transcriptional processes in other clinically important viruses.

**Impact Statement**

Hepatitis B virus (HBV) is a major global health concern, causing 1.1 million deaths in 2022 and 1.2 million new infections each year. It is a leading cause of serious liver conditions, including cirrhosis and liver cancer. Although vaccines can prevent HBV infection, there is currently no cure. HBV produces different types of genetic messages (RNAs), including spliced versions that are processed by the host cell’s machinery. These spliced RNAs help the virus evade the immune system and make it harder for treatments to fully clear the infection. In this study, we analyzed 279 HBV samples from liver tissues and laboratory-grown cells and found that the efficiency of host-mediated splicing of viral RNA reflects the severity of disease. Using advanced artificial intelligence tools, we mapped the splicing patterns in both the virus and human host and investigated over 4,700 HBV genomes. We discovered that key HBV splice donor sites are highly conserved and resemble host splice donor sites, whereas acceptor sites are more cryptic, suggesting the virus has evolved RNA sequences that are compatible with the host cell’s splicing machinery while accommodating its compact genome. Insights into this viral adaptation may help researchers identify new biomarkers for disease severity and develop therapeutic strategies that disrupt the virus’s ability to exploit the host cell’s machinery.

## Data Summary

The raw RNA-sequencing (RNA-seq) libraries analyzed in this study were previously published and are available in the Gene Expression Omnibus under accession number GSE155983. These libraries form part of a curated collection of 279 RNA-seq datasets derived from HBV-associated liver biopsy tissues and cultured cells. Further details and metadata are available in the associated GitHub repository (https://github.com/lcscs12345/HBV_splicing_paper_2025) and previously published supplementary data (https://pmc.ncbi.nlm.nih.gov/articles/instance/8115900/bin/mgen-7-492-s002.xlsx).

## Introduction

Chronic hepatitis B virus (HBV) infection remains a major global health challenge and the leading cause of hepatocellular carcinoma (HCC) [1]. Current antiviral therapies suppress viral replication rather than eliminate the virus, allowing it to persist in infected hepatocytes. HBV continues to drive nearly half of all HCC cases worldwide, with the Asia-Pacific region bearing a disproportionate burden, accounting for over 70% of HBV-related HCC deaths [2,4]. With liver cancer incidence projected to exceed one million cases annually by 2025 [1], there is an urgent need to better understand the viral sequence determinants that contribute to disease progression and treatment resistance to inform precision strategies for monitoring and intervention.

**Fig. 1.**
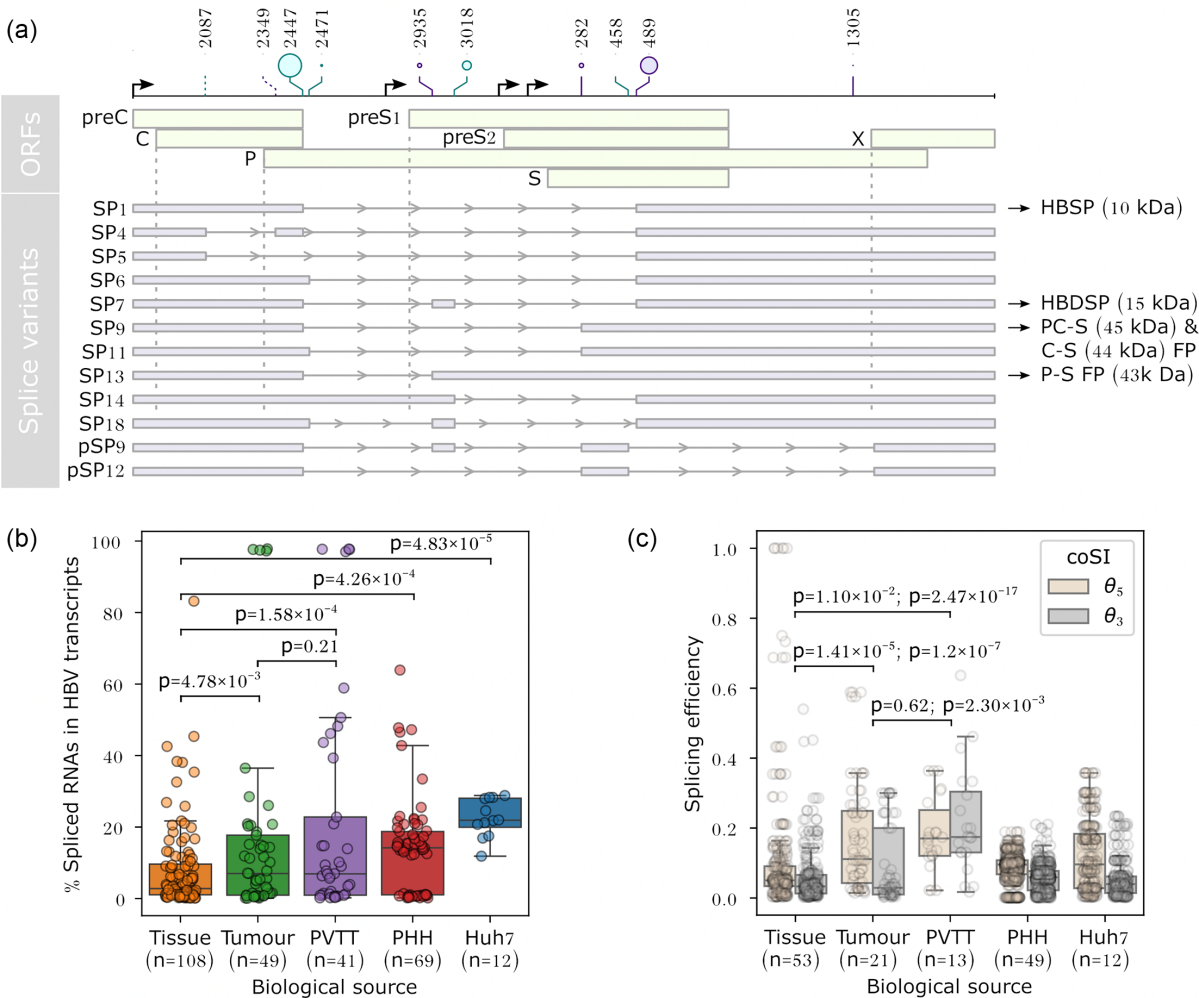
The proportion of spliced HBV RNAs varies across liver tissue types and disease progression. (**a**) Representative splice variants identified in 279 HBV transcriptomes. All splice sites are located within the ORFs. Solid lines indicate more common splice sites, while dotted lines mark less common splice sites. Lollipop sizes represent splicing efficiency, scaled by the median coSI (range 0.0060–0.21). Protein isoforms derived from these splice variants include HBSP (10 kDa), HBDSP (15 kDa) and fusion proteins such as PC–S (45 kDa), C–S (44 kDa) and P–S (43 kDa). (**b**) Liver tissues adjacent to tumours show the lowest proportions of spliced HBV RNAs, followed by tumours, advanced-stage PVTT, HBV-infected PHHs and HBV-transfected Huh7 cells. *P* values were derived from one-sided t-tests for independent samples. (**c**) Across splice variants, donor sites generally exhibit higher splicing efficiency than acceptor sites, as indicated by higher coSI θ_5_ scores compared to θ_3_ scores.

HBV is a DNA retro-transcribing virus with a compact 3.2 kb genome that encodes four canonical ORFs responsible for producing the major viral proteins: core (C), polymerase (P), surface (S) and X. Embedded within these ORFs are multiple splice donor and acceptor sites in the pregenomic RNA (pgRNA) [5,7]. Alternative usage of these splice sites generates ~20 distinct spliced RNA variants, which in turn give rise to a variety of novel protein isoforms with diverse functions (Fig. 1a). For example, the hepatitis B spliced protein (HBSP), derived from the SP1 splice variant, promotes immune escape by reducing immune cell recruitment and suppresses apoptosis via PI3K/Akt activation [8,9]. In contrast, hepatitis B doubly spliced protein (HBDSP), derived from SP7, functions as a transactivator that promotes apoptosis in HCC cells [10,11]. The precore–surface (PC–S) and core–surface (C–S) fusion proteins derived from SP9 are factors that regulate viral replication [12]. The polymerase–surface fusion protein (P–S), derived from SP13, is a structural protein that may substitute for the large HBV surface protein [13]. The viral oncogenic transactivator HBx, encoded by an intact X ORF retained in most HBV splice variants, plays a key role in transcriptional regulation and hepatocarcinogenesis [5].

Notably, HBV splice variants can be packaged into defective viral particles and detected in patient sera [14,17]. Although these particles are incapable of autonomous replication, elevated levels of HBV splicing in serum correlate with progression of chronic liver disease [15,16]. Such splicing activity can be observed years before HCC diagnosis, even in patients with normal alpha-fetoprotein tumour marker levels [17]. These observations support the potential use of HBV splice variants as biomarkers of disease progression.

HBV is classified into at least ten genotypes (A–J), each with distinct geographic distributions and sequence characteristics that affect clinical outcomes [18,20]. These genotypes differ in their association with disease progression, treatment response and risk of HCC. We previously demonstrated that the major HBV genotypes express markedly distinct compositions of splice variants [6], suggesting that regulation of splicing may be genotype-specific. HBV splicing can be regulated both *in cis*, for example, by the post-transcriptional regulatory element [21], and *in trans*, through host factors such as DDX5 and DDX17 [22]. A recent study showed that DDX5 and DDX17 RNA helicases can suppress recognition of an HBV splice donor site [22]. However, the sequence determinants and genotype-specific features that control splice site selection and usage in HBV remain poorly understood, particularly in the context of disease progression and genotype-specific variation.

In this study, we employ large language and deep learning models (SpliceBERT and OpenSpliceAI) [23,24] to systematically analyse HBV splice site usage across 279 curated viral transcriptomes [6] and 4,706 viral genomes from HBVdb [20]. Our findings reveal that HBV splicing is shaped by sequence context, host-like features and viral genotype, offering insights into potential implications for viral replication, immune evasion, oncogenesis and biomarker development for precision strategies.

## Methods

### Data

We curated a total of 279 HBV transcriptomes from our previous transcriptomic study [6]. The dataset includes BAM and SJ.out.tab files generated by STAR v2.7.6a in 2-pass mode [25] and Gene Transfer Format (GTF) annotation files produced by StringTie v1.3.3b [26]. For *Homo sapiens*, UCSC Genome Browser’s hg19 genome and GENCODE annotation v41lift37 were used [27,28].

### Splicing efficiency analysis

Splicing efficiency scores for donor and acceptor sites [completed splicing index (coSI) θ5 and θ3 scores, respectively] were computed from BAM files using IPSA [29]. To map splice variant IDs to their respective coSI scores, GTF files were converted to BED file format using UCSC Genome Browser's KentUtils and intersected with IPSA outputs using BEDTools v2.31.1 [30].

### Embedding-based clustering

To capture conserved splicing patterns across eukaryotic species, we fine-tuned SpliceBERT using the Spliceator training set, generating model weights for donor and acceptor sites [24, 31]. We modified SpliceBERT fetch_embedding.py to incorporate fine-tuned weights into the pretrained 510 nt model using HuggingFace Transformers v4.27.2 [32]. Input files were prepared by generating HBV pgRNA consensus sequences from BAM files using BCFtools v1.22 [33]. Splice junctions supported by ≥5 uniquely mapping reads were extracted from SJ.out.tab files and converted to BED format.

For *H. sapiens*, approximately half of the splice sites were extracted to streamline the analysis. Non-donor GU sites and non-acceptor AG sites within protein-coding regions were used as negative controls. Viral and host data were combined, and the final layer of nucleotide embeddings was extracted for all splice sites, representing local sequence context. All model inference and embedding extraction were performed on NVIDIA A100 40G GPUs.

### Gene ontology analysis

Gene ontology analysis was conducted on a subset of host splice sites used for the embedding-based clustering analysis. We excluded those located in the 5′ leader and 3′ trailer sequences to focus on coding sequence (CDS) splice sites. Next, we identified splice donor and acceptor sites that co-occur within the same CDS using BEDTools intersect [30]. This filtering resulted in 586 unique gene IDs, which were used as input for g:Profiler e113 with default settings [34].

### Splice site classification

To classify HBV splice sites, we analyzed 4,706 HBV genomes obtained from HBVdb [20]. All genomes selected for this analysis were of the same length to prevent misalignment at splice sites. These genomes consisted of genotypes B, C, F and recombinant forms (RFs), as most sequences within these genotypes share similar genome sizes, enabling tractable and comparable splice site motif analysis. Genotype C is clinically associated with worse prognosis, while genotype B is linked to younger patients with non-cirrhotic liver [35], and together these genotypes represent about one-third of HBVdb entries.

Genomic sequences were rearranged to match the structure of pgRNA and extended with 200 nt flanking regions upstream of the preC start codon and downstream of the X stop codon to provide biological context and minimize artificial sequence padding. We applied a sliding window of 400 nt with 1 nt overlap across each genome. Each window was tokenized and passed to the fine-tuned SpliceBERT model for inference. For each window, the sequence-level representation was extracted from the final hidden state of the [CLS] token (a 512-dimensional vector) and used to predict splicing propensity for HBV splice sites versus non-splice sites.

We also applied a similar sliding window approach for splice site classification using OpenSpliceAI v0.0.4 [23], with the 400 nt flanking-size model trained on *H. sapiens* MANE transcript annotation [36]. For visual comparison, splice sites were grouped into higher usage (coSI≥0.1) and lower usage (coSI<0.1) categories.

### Sequence conservation analysis

To assess splice site conservation, we first calculated the proportion of genomes that share the same HBV splice donor and acceptor dinucleotides (GU and AG, respectively). For comparison, we also included non-splice site GU and AG positions as controls.

To visualize the splice site contexts, we extracted the flanking sequences around splice sites and plotted the base frequencies using kpLogo v1.1 [37]. To quantify the similarity between HBV and host splice site contexts, we performed motif similarity analysis using Tomtom implemented in memelite v0.2.0 [38,39]. Individual HBV splice site sequences (30 nt) were one-hot encoded and compared against the position weight matrices derived from host’s typical and CDS splice sites. These position weight matrices were generated from splice site sequences (30 nt) using Biopython [40].

### Statistical analysis and data visualization

All data processing and statistical analyses were performed using pandas v2.2.2 [41] and NumPy v1.26.4 [42]. Normalized mutual information (NMI) scores were calculated using scikit-learn v1.6.1 [43] to quantify the similarity between clustering results and known splice site labels. Model performance metrics, including area under the receiver operating characteristic curve (AUROC), area under the precision-recall curve (AUPRC), F1 score, precision and recall, were also computed using scikit-learn. These metrics were used to evaluate the ability of SpliceBERT and OpenSpliceAI to distinguish HBV splice sites from non-splice sites across curated genomic datasets. Independent t-tests, Mann–Whitney U tests, Wilcoxon signed-rank tests and Spearman’s correlation analyses were conducted using SciPy v1.13.1 [44] to assess statistical significance and relationships among the proportion of spliced HBV RNAs, splicing efficiency scores and clustering patterns. All visualizations were generated using Matplotlib v 3.6.3 [45] and seaborn v0.13.2 [46].

### Code and data availability

Jupyter notebooks, scripts and processed data for the study can be found at https://github.com/lcscs12345/HBV_splicing_paper_2025.

## Results

### Splicing patterns in HBV transcriptomes reflect disease state

We previously assembled 279 HBV transcriptomes by analysing 513 publicly available RNA-sequencing (RNA-seq) libraries derived from HBV-associated liver biopsy tissues and cultured cells [6]. These transcriptomes revealed highly complex splicing patterns across the major HBV genotypes [6] (Figs 1 and S1, available in the online Supplementary Material). Notably, all HBV splice sites identified span protein-coding regions (CDS splice sites).

To assess the clinical relevance of HBV splicing, we compared the proportions of spliced HBV RNAs across different sample types. The median proportion of spliced HBV RNAs ranged from 3 to 22%. Advanced-stage HCC samples, as characterized by portal vein tumour thrombosis (PVTT), and earlier-stage tumours exhibited significantly higher levels of HBV splicing than adjacent non-tumour tissues (Fig. 1b, one-sided t-test for independent samples, *P* values=1.58×10^−4^ and 4.78×10^−3^, respectively). Furthermore, genotype C-positive PVTT samples exhibited elevated levels of HBV splicing compared to genotype B (Fig. S1A, one-sided t-test for independent samples, *P* value=1.28×10^−2^), consistent with previous reports linking genotype C to worse prognosis [35]. Most HBV-infected primary human hepatocytes (PHHs) and HBV-transfected Huh7 hepatoma cells also showed elevated splicing levels compared to adjacent non-tumour tissues (Fig. 1b, one-sided t-test for independent samples, *P* values=4.26×10^−4^ and 4.83×10^−5^, respectively), likely influenced by experimental conditions, such as the use of a strong cytomegalovirus (CMV) promoter for expressing HBV pgRNAs [6]. Consistent with this, PHH and Huh7 cells exhibited a greater diversity of splice variants (Table S1). In particular, Huh7 cells showed a high proportion of SP1 (~12%), which further contributes to their overall elevated splicing levels (Fig. S1B).

Further analysis of HBV transcriptomes revealed that tumour tissues exhibited significantly higher splicing efficiency than adjacent non-tumour tissues, as measured by the coSI [29,47] (Fig. 1c, one-sided t-tests for independent samples, *P* values=1.41×10^−5^ and 1.20×10^−7^ for θ_5_ and θ_3_, respectively). PVTT samples showed even greater splicing efficiency than tumours (Fig. 1c, one-sided t-tests for independent samples, *P* values=0.62 and 2.30×10^−3^ for θ_5_ and θ_3_, respectively), whereas the overall HBV splicing levels did not differ significantly (Fig. 1b, one-sided t-test for independent samples, *P* value=0.21). These findings indicate that coSI captures differences across disease stages. This suggests that the splice site-level measurement may provide greater discriminatory power for distinguishing disease stages (adjacent non-tumour tissue, tumour tissue and PVTT samples) than the overall proportion of HBV spliced RNAs, a metric that has been proposed as an emerging biomarker [16]. Although HBV splice sites are generally weak, the most commonly expressed splice variants were derived from donor sites with higher splicing efficiency than acceptor sites, with the exception of SP6 (Figs 1c and S1C, D).

### HBV splice site features are less distinct than host splice sites

**Fig. 2.**
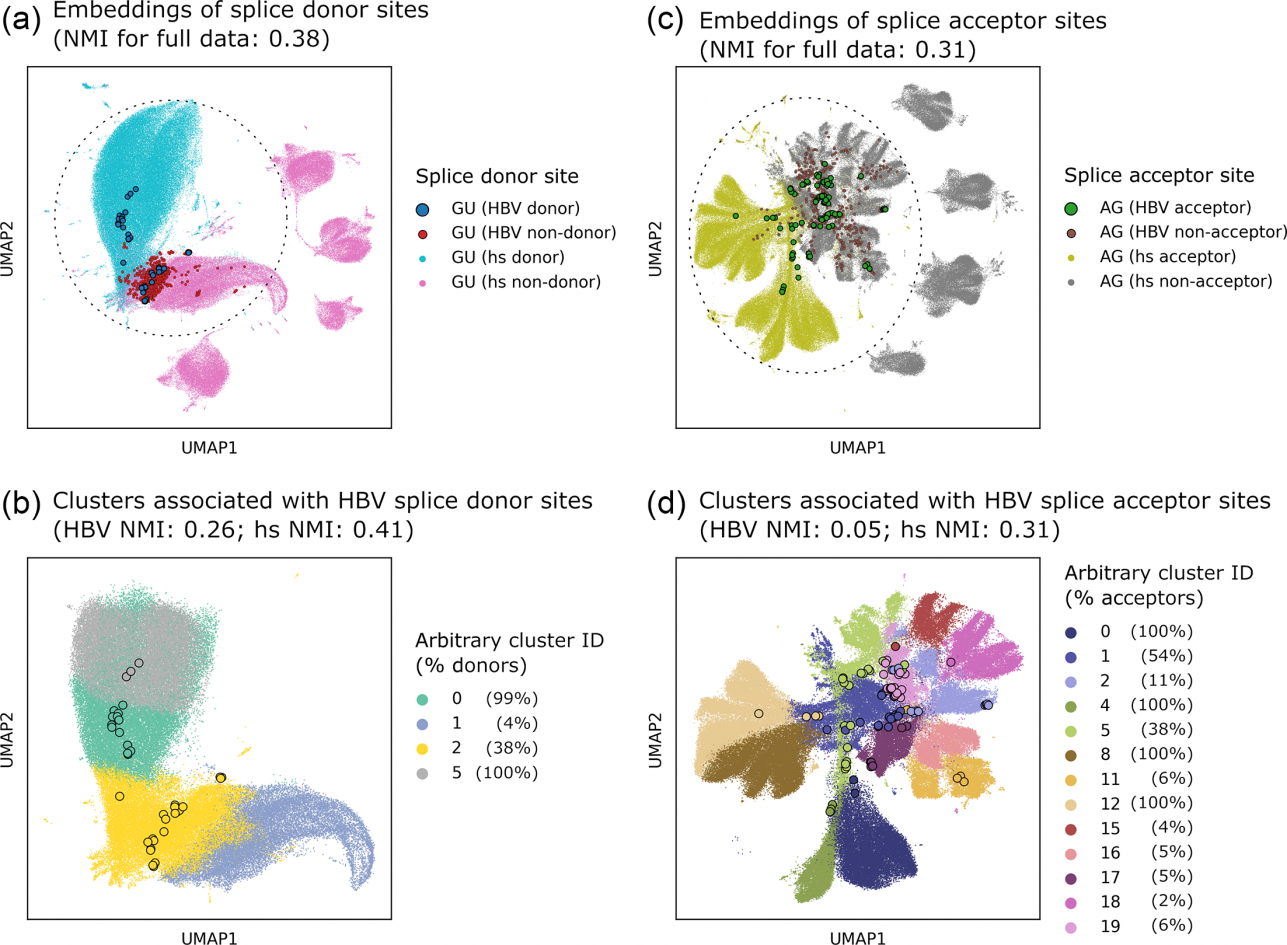
Most HBV splice sites are distinct from host splice sites. Clustering of nucleotide embeddings from HBV and *H. sapiens* (hs) (**a and b**) splice donor and (**c and d**) acceptor sites, extracted from the final layer of SpliceBERT models. These models were fine-tuned using a subset of the Spliceator training set with all *H. sapiens* splice sites removed. Points for HBV splice sites are outlined in black. (**a and c**) Marker size indicates different groups of sites: largest for HBV donor sites, medium for HBV non-donor sites and smallest for host donor and non-donor sites. Embeddings were subjected to dimensionality reduction using PCA, followed by UMAP and clustered using the Leiden algorithm. Panels (b) and (d) show clusters highlighted by dotted circles in (a) and (c), respectively. NMI scores in these panels indicate that HBV splice sites are more distinct from host splice sites. This analysis includes 41 HBV donor and 94 acceptor sites, along with 627 non-donor GU sites and 529 non-acceptor AG sites, derived from 4 randomly selected HBV transcriptomes among HBV-associated samples. Host splice sites include 95,323 donor and 99,179 acceptor sites, along with 80,542 non-donor GU sites and 123,162 non-acceptor AG sites. See also Fig. S2.

As multiple lines of evidence suggest that HBV leverages the host splicing machinery during cancer progression, we sought to decipher and compare the splicing code between the virus and host. To decode the viral and host splicing patterns, we harnessed the representational power of SpliceBERT, the state-of-the-art transformer-based large language model (LLM) [24]. We fine-tuned two versions of SpliceBERT: one using the full Spliceator training set [31] to capture conserved splicing patterns across 114 species, a strategy shown to outperform models trained solely on *H. sapiens* data [24], and another with all *H. sapiens* splice sites removed (Figs 2 and S2, respectively). The latter ensures that the datasets for fine-tuning and test sets do not overlap. We then compared HBV splice sites (41 donor and 94 acceptor sites from four representative transcriptomes) with randomly selected host splice sites (95,323 donor and 99,179 acceptor sites) by extracting nucleotide embeddings and applying principal components analysis (PCA), uniform manifold approximation and projection (UMAP) and Leiden hierarchical clustering.

We first computed three NMI scores: one for the full dataset and two for regions where both HBV and host splice sites occur in the shared embedding space (Figs 2 and S2). These shared regions enable direct comparisons between HBV and host splice sites. While host splice sites and non-splice sites (non-donor GU and non-acceptor AG dinucleotides) formed distinct clusters, HBV splice sites were distributed across multiple clusters and exhibited NMI scores 2–6 times lower than those of host splice sites (Fig. 2b, d).

**Fig. 3.**
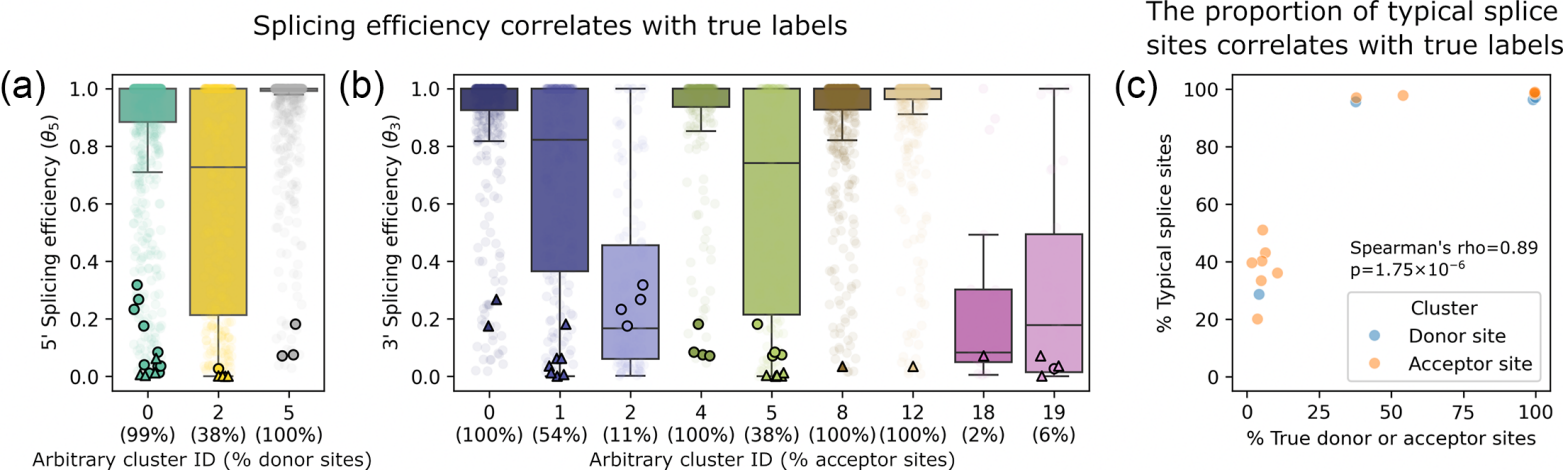
Splicing efficiency and the proportion of typical splice sites correlate with true label proportions in Leiden clusters. (a and b) Points representing HBV splice sites are outlined in black, where circles and triangles indicate well-known and novel HBV splice sites, respectively. Leiden clusters were derived from the embedding space of splice sites shown in Fig. 2, where HBV and host splice sites were grouped using nucleotide embeddings from SpliceBERT. Clusters enriched for true splice sites (HBV and host) tend to show higher splicing efficiency (Spearman’s correlation=0.81, *P* value=2.51×10^−3^) and a greater proportion of typical host splice sites (Spearman’s correlation=0.89, *P* value=1.75×10^−6^). (c) Percent typical host splice sites were calculated as the proportion of splice sites spanning the boundaries of exons and non-coding regions (GU/AG dinucleotides). See also Figs S2 and S3.

To determine whether this weaker alignment between true labels and cluster assignments was driven by well-known SP variants or more recently discovered novel variants (Fig. 1a, e.g. pSP variants) [6,48], we computed NMI scores for these groups separately. Donor sites from SP variants showed higher clustering similarity compared to novel variants (Table 1, NMI=0.46 and 0.07, respectively) and host transcripts (Fig. 2b, NMI=0.41). This indicates that well-known HBV donor sites formed clusters enriched for splice sites, whereas many novel donor sites formed mixed clusters containing both splice and non-splice sites (Figs 2b, 3a, S2B and S3A). In contrast, HBV acceptor sites (including those from SP variants) showed low NMI scores (Table 1, 0.04–0.05), suggesting that HBV acceptor sites are generally less distinct in the embedding space (Figs 2d, 3b, S2D and S3B). These unexpected patterns led us to hypothesize that a set of HBV splice sites, in particular those located in these mixed clusters, may be less efficiently recognized by the splicing machinery, as indicated by their cryptic sequence contexts.

**Table 1.** NMI scores between Leiden clusters of HBV splice and non-splice sites.

Fine-tuning set	Composition of clusters	Donor and non-donor NMI	Acceptor and non-acceptor NMI
Without host*	SP and novel	0.26	0.05
	SP	0.46	0.04
	Novel	0.07	0.04
Full†	SP and novel	0.13	0.05
	SP	0.18	0.07
	Novel	0.04	0.03

Note: SP refers to HBV splice sites from SP variants; novel refers to HBV splice sites from novel variants.

* Spliceator training set without *H. sapiens*. See the related Figs 2 and 3.

† Full Spliceator training set (114 species). See the related Figs S2 and S3.

To test this hypothesis, we mapped experimentally determined splicing efficiency scores (coSI) to the Leiden clusters. To assess how well clusters capture true splice sites, we calculated the proportion of true donor or acceptor splice sites from HBV and host within each cluster (e.g. if a cluster contains 100 donor site candidates and 60 of them are annotated as true donor sites, the percentage is 60%). We found that splicing efficiency positively correlated with the proportion of true, annotated splice sites within each cluster (Fig. 3a, b, Spearman’s correlation=0.81, *P* value=2.51×10^−3^). This supports our idea that splice sites which are more efficiently recognized and processed by the splicing machinery tend to cluster together, while weakly recognized sites (especially acceptor sites) are more likely to co-cluster with non-splice sites. Notably, although well-known HBV donor sites appear in clusters enriched for highly efficient sites (Fig. 3a, cluster IDs 0 and 5), many well-known HBV acceptor sites are found in mixed clusters containing non-acceptor sites (Fig. 3b, cluster IDs 2 and 5).

We further examined the distribution of typical host splice sites that span the boundaries of exons and non-coding regions across these clusters. Consistent with the above results, clusters with higher proportions of true, annotated splice sites also contained more typical splice sites (Fig. 3c, Spearman’s correlation=0.89, *P* value=1.75×10^−6^). In contrast, CDS splice sites, which are embedded within protein-coding regions, are more likely to fall into mixed clusters and have a lower splicing efficiency. This supports the idea that typical splice sites are more efficiently processed, while CDS splice sites are more likely to evade recognition due to their cryptic sequence contexts.

These findings suggest that HBV’s compact genome imposes structural constraints, requiring it to accommodate overlapping coding regions and essential RNA elements. To maintain functionality under these constraints, HBV appears to exploit host-like motifs that are suboptimal yet spliceable, enabling splicing without disrupting its gene structure. A similar configuration was observed in a small subset of host genes, comprising only 3 and 1% of all CDS splice donor and acceptor sites, respectively. This subset was enriched for the Gene Ontology Biological Process term ‘organelle organization’ (GO:0006996; adjusted *P* value=10^−7^; Fig. S4).

### AI models reveal genotype-specific splicing patterns

Our analyses suggest that splice site recognition by the host splicing machinery is highly context-dependent. However, it remains unclear whether HBV splice sites are shaped by genotype-specific sequence features and whether these features correlate with splice site usage. Since our goal is to predict HBV splice sites in a host context, we used models fine-tuned on the full Spliceator training set for this analysis. This approach ensures that the models have learnt host splicing patterns that may be biologically relevant.

To classify HBV splice sites from non-splice sites, we performed a sliding window analysis across 4,706 HBV genomes, including 1,728 genotype B, 2,084 genotype C, 252 genotype F and 643 RFs [20]. Each 400 nt window was scored using SpliceBERT and OpenSpliceAI, the latter being a deep convolutional neural network trained using *H. sapiens* data [23]. Although these models were trained for binary classification, we grouped HBV splice sites into higher and lower usage categories based on coSI scores.

**Fig. 4.**
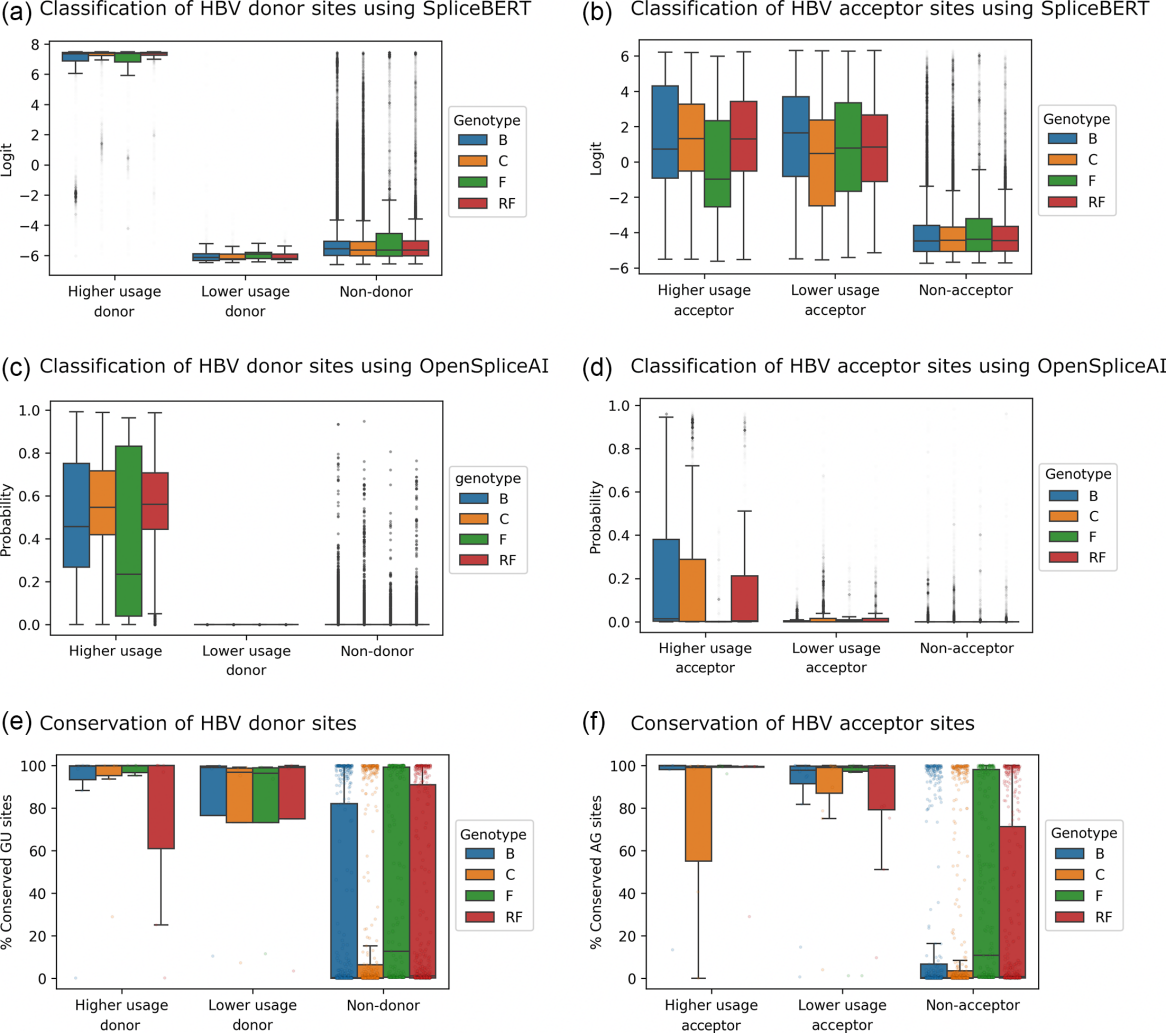
HBV splice donor and acceptor sites are conserved and distinct from non-splice sites. Splice sites were categorized by usage level based on coSI scores (≥0.1 for higher usage, <0.1 for lower usage). Higher usage splice sites exhibit higher (a and b) SpliceBERT [CLS] scores (logit values) and (c and d) OpenSpliceAI prediction probabilities. In contrast, lower usage splice sites generally show low scores similar to non-splice sites, except for SpliceBERT scores at lower usage acceptor sites. (e and f) Higher usage splice sites also exhibited the least variable GU/AG dinucleotide frequencies, followed by lower usage splice sites and non-splice sites. Bootstrap resampling (10,000 iterations) showed that higher and lower usage splice site dinucleotides are similarly conserved, with near zero 95% confidence intervals for donor sites (B: 0.06–0.22; C: 0.10–0.14; F: 0.01–0.27; RF: −0.14–0.14) and acceptor sites (B: −0.08–0.02; C: −0.14–0.02; RF: −0.01–0.06; F: −0.02–0.19). Sites were selected from a diverse set of HBV genomes, comprising 1,728 genotype B, 2,084 genotype C, 252 genotype F and 642 RF. Collectively, a genotype may habor up to 7 higher usage and 4 lower usage donor sites, and 6 higher usage and 12 lower usage acceptor sites.

Higher usage splice sites consistently scored higher than lower usage and non-splice sites (Fig. 4), indicating that more frequently used sites possess more recognizable sequence features. This pattern aligns with the embedding-based clustering analysis, which also showed that higher usage splice sites, particularly HBV donor sites, cluster more distinctly than lower usage sites (Figs 2, 3, S2 and S3 and Table 1). However, distinguishing lower usage splice sites, particularly donor sites, proved more challenging. This difficulty was reflected in both models, in conjunction with notable differences in scoring across HBV genotypes. Prediction scores for donor splice sites varied substantially between genotypes, suggesting that genotype-specific sequence variation affects how well these sites are scored by artificial intelligence (AI) models trained on *H. sapiens* or multi-species splicing data. SpliceBERT performed better for acceptor site prediction, while OpenSpliceAI showed stronger performance for donor sites (Table 2), highlighting the complementary strengths of these models.

**Table 2.** Performance of AI models in predicting HBV splice sites. Metrics include AUROC, AUPRC, F1 score, precision and recall, evaluated using curated HBV genomic datasets. See related Figs4  5.

AI model		AUROC	AUPRC	F1 score	Precision	Recall
SpliceBERT	Donor	0.72	0.59	0.52	0.47	**0.59**
Acceptor	**0.90**	**0.60**	**0.60**	0.65	**0.56**
OpenSpliceAI	Donor	**0.78**	**0.61**	0.51	**0.99**	0.34
Acceptor	0.89	0.57	0.13	**0.97**	0.07

Note: The best results are marked in bold. This analysis includes 40,117 donor sites, 757,682 non-donor GU sites, 67,262 acceptor sites and 652,160 non-acceptor AG sites, derived from 2,084 genotype C, 1,728 genotype B, 252 genotype F and 642 RFs.

**Fig. 5.**
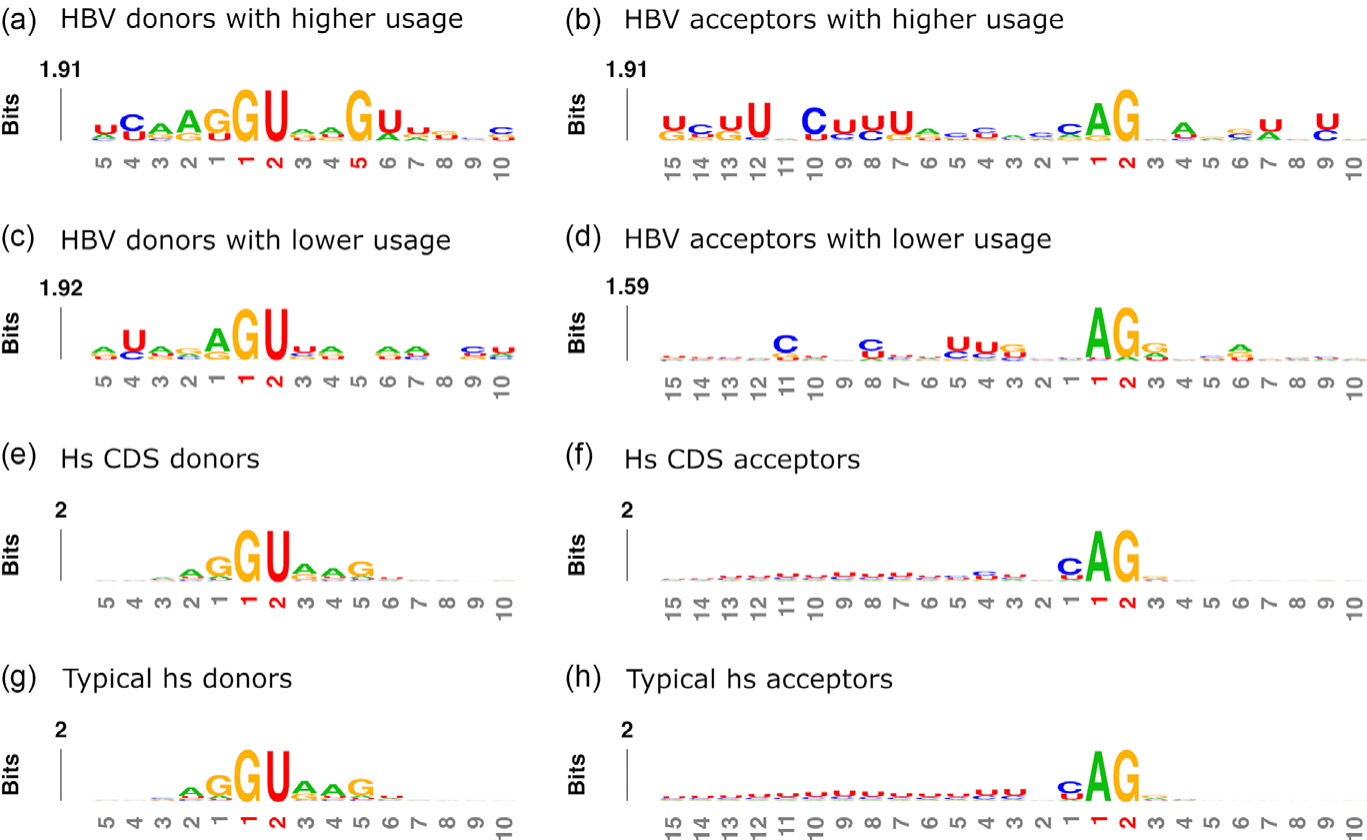
Sequence contexts of HBV and host splice sites. (a and c) Base frequencies illustrate HBV donor sites with higher and lower usage (both 18,828 sites), (b and d) HBV acceptor sites with higher and lower usage (23,535 and 51,777 sites, respectively), (e and g) host’s CDS and typical donor sites (1,243 and 43,004), (f and h) host’s typical acceptor sites (646 and 47,428 sites, respectively).

Although these models were not trained on viral data, the higher scores assigned to more frequently used HBV splice sites suggest that AI models can identify functional sequence features resembling host splice sites. This capability enables us to uncover genotype-specific splicing patterns. Notably, the elevated SpliceBERT scores for higher usage splice sites in genotype C align with the more extensive splicing observed in liver issues infected with this genotype (Figs 4a, b and S1A). While the development of virus-specific models may improve predictive accuracy, the priority remains experimental validation to establish the clinical relevance and therapeutic potential of these predictions.

### HBV splice sites are conserved

As HBV splice site usage varies across genotypes and higher usage sites tend to be more distinguishable by AI models, we next asked whether these frequently used splice sites are also more conserved across HBV genomes. If so, this would suggest that higher usage splice sites are functionally important and subject to selective pressure, distinguishing them from lower usage and non-splice sites.

To test this idea, we computed the proportion of canonical splice site dinucleotides (GU for donor sites and AG for acceptor sites) across 4,706 HBV genomes. HBV splice sites are conserved, with higher usage splice sites showing the least variable dinucleotide frequencies, followed by lower usage splice sites and non-splice sites (Fig. 4e, f). This variability reflects differences across splice site positions rather than individual genomes, as we collapsed splice sites into unique positions per genotype to compute proportions (Fig. 4e, f). Bootstrap resampling (10,000 iterations) confirmed that differences in dinucleotide conservation between higher and lower usage splice sites are negligible (Fig. 4e, f).

**Fig. 6.**
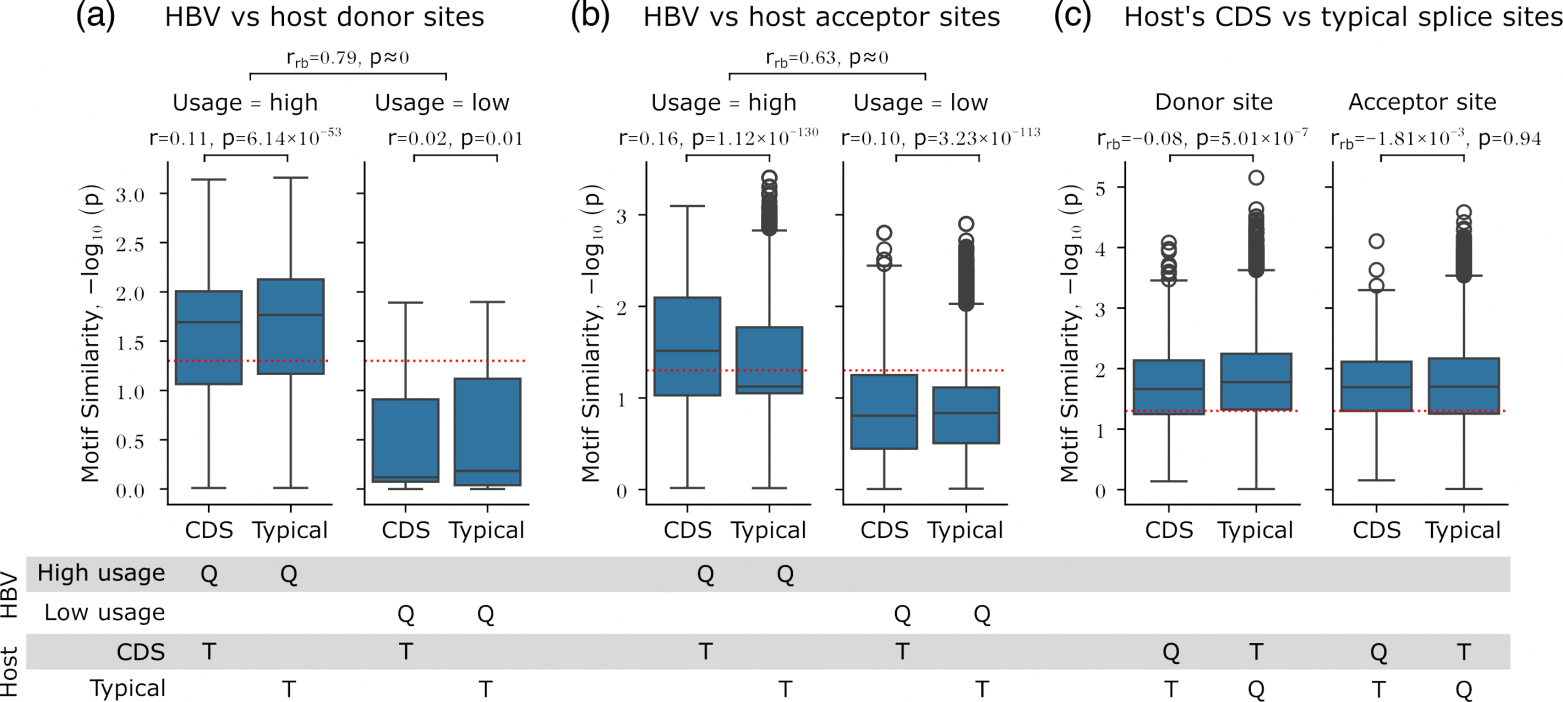
Higher usage HBV splice sites show greater similarity to host splice sites than lower usage sites. Motif similarity was assessed using Tomtom, comparing the sequence contexts of individual HBV splice sites to host CDS and typical splice sites. Values are shown as −log10(p), where higher values indicate greater similarity; values above the dotted lines correspond to *P* value<0.05. ‘Q’ indicates one-hot encoded queries; ‘T’ indicates a position weight matrix target. (**a**) For donor sites, higher usage sites were slightly but significantly more similar to host’s typical donor sites than to CDS donor sites (two-sided Wilcoxon signed-rank test, effect size *r*=0.11, *P* value=6.14×10^−53^). (**b**) For acceptor sites, higher usage sites were slightly but significantly more similar to host’s CDS acceptor sites than to typical acceptor sites (two-sided Wilcoxon signed-rank test, effect size *r*=0.16, *P* value=1.12×10^−130^). Differences between higher usage HBV-host and lower usage HBV-host splice site contexts were assessed using two-sided Mann–Whitney U tests and rank-biserial correlation (*r*_rb_). (**c**) Host’s CDS and typical splice site contexts were compared as controls.

To investigate sequence contexts beyond dinucleotides, we analyzed base frequencies around HBV donor and acceptor sites grouped by usage levels (Fig. 5). For visual comparison, we included host splice sites located within protein-coding regions (CDS splice sites) and those that span the boundaries of exons and non-coding regions (typical splice sites). Next, we compared the sequence contexts of individual HBV splice sites to host CDS and typical splice sites using Tomtom [38,39] (Fig. 6).

Although all HBV splice site dinucleotides are conserved (Fig. 4e, f), the sequence contexts of higher usage splice sites were significantly more similar to host than lower usage sites (Fig. 6, two-sided Mann–Whitney U tests, rank-biserial correlations *r*_rb_=0.79 and 0.63 for donor and acceptor site comparisons, respectively, *P* values≈0). Higher usage donor sites exhibited a slight but significant similarity to typical host donor sites (two-sided Wilcoxon signed-rank test, effect size *r*=0.11, *P* value=6.14×10^−53^), whereas higher usage acceptor sites showed a slight but significant similarity to host CDS acceptor sites (two-sided Wilcoxon signed-rank tests, effect size *r*=0.16, *P* value=1.12×10^−130^). These findings indicate that the sequence contexts of higher usage HBV splice sites are more conserved and show a stronger signal of viral–host coevolution than lower usage splice sites, consistent with AI model predictions (Figs 2–4, S2 and S3 and Table 1). In contrast, lower usage sites appear more cryptic despite retaining canonical GU/AG dinucleotides. The mechanisms by which HBV exploits these distinct splice site properties warrant further investigation.

## Discussion

Our findings demonstrate that HBV splicing is not co-transcriptional noise but a regulated process with functional consequences in disease progression. We showed that HBV splicing levels are elevated in liver tumours and advanced-stage PVTT samples (Figs 1 and S1), consistent with a role in hepatocarcinogenesis. Previous studies have shown that these splice variants can be packaged into defective virus particles [14,17]. These defective particles are detectable in patient sera, and their overall proportion has been proposed as an emerging biomarker [16].

The splice site-level information shown in this study may act as a mechanistically informative biomarker with greater discriminatory power. For example, splicing efficiency at the acceptor sites could be used to distinguish between PVTT samples and tumours (Fig. 1c). Furthermore, SP13 is more prevalent in PVTT compared to tumours, whereas SP1 is more prevalent in adjacent non-tumour tissues compared to PVTT (Fig. S1B). Future work could validate whether a panel of splice variants correlates with clinical outcomes such as PVTT occurrence or reduced likelihood of functional cure. These HBV splice variants and their encoded proteins (Fig. 1a) have also been shown to control viral replication dynamics [12], suppress host innate immune response, such as IFN-*α* signalling [49], and are associated with a reduced chance of achieving functional cure [15]. A functional cure is a clinical endpoint where there is a sustained loss of hepatitis B surface antigen and HBV DNA after a finite course of treatment [50].

To dissect the sequence-function relationship of HBV splice sites, we leveraged 279 HBV transcriptomes derived from deep RNA-seq datasets [6], 4,706 HBV genomes curated from HBVdb [20] and state-of-the-art AI models, including SpliceBERT LLM [24] and OpenSpliceAI deep learning framework [23]. This integrative approach enabled us to identify splice sites with high usage and predictive sequence features.

We found that HBV splice sites are generally conserved. Higher usage splice sites are more readily recognized by AI models and share sequence characteristics with host splice sites, despite the constraint imposed by the virus’s compact genome. These converging lines of evidence suggest that HBV may co-opt host-like sequence motifs to regulate splicing in a genotype-dependent manner, thereby enhancing its persistence and immune evasion [7].

Collectively, our study provides a comprehensive framework for understanding HBV splicing through the lens of over 50,000 years of virus–host coevolution [18,51, 52]. By applying cutting-edge computational tools to large-scale genomic and transcriptomic data, we highlight the conserved, mechanistically important and clinically relevant nature of HBV splicing. These findings lay the groundwork for precision therapeutic targeting of HBV splice variants and their regulatory elements, offering new avenues for intervention in chronic HBV infection and HBV-associated HCC, including the development of novel clustered regularly interspaced short palindromic repeats (CRISPR)-based therapies [53]. Furthermore, our findings demonstrate the broader utility of AI models in decoding viral genomic architectures and regulatory mechanisms, with implications for studying splicing and post-transcriptional control in other clinically important viruses.

## Supplementary material

10.1099/mgen.0.001616 Supplementary Material 1.

## Abbreviations

AI: artificial intelligence
AUPRC: area under the precision-recall curve
AUROC: area under the receiver operating characteristic curve
CDS: coding sequence
CMV: cytomegalovirus
coSI: completed splicing index
CRISPR: clustered regularly interspaced short palindromic repeats
C–S: core–surface
GTF: Gene Transfer Format
HBDSP: hepatitis B doubly spliced protein
HBSP: hepatitis B spliced protein
HBV: hepatitis B virus
HCC: hepatocellular carcinoma
LLM: large language model
NMI: normalized mutual information
PCA: principal components analysis
PC–S: precore–surface
pgRNA: pregenomic RNA
PHH: primary human hepatocyte
P–S: polymerase–surface
PVTT: portal vein tumour thrombosis
RF: recombinant form
RNA-seq: RNA-sequencing
UMAP: uniform manifold approximation and projection
