# Supplementary material for: Decoding the interconnected splicing patterns of hepatitis B virus and host using large language and deep learning models
Source: Microb Genom. 2026 Jan 16;12(1):001616. doi: 10.1099/mgen.0.001616 (PMC12811152; doi:10.1099/mgen.0.001616)
Supplement: Supplementary Material 1. [file mgen-12-01616-s001.pdf]

SUPPLEMENTARY MATERIALS

Table S1. Statistics of HBV splice variants in various biological sources.

**Biological source**	**% HBV RNA**	**Average number of splice variants**	**Number of exons**
Tissue	1.8	7.1	2.3
Tumour	5.8	3.5	2.2
PVTT	9.7	2.2	2.1
PHH	1.7	11.6	2.4
Huh7	1.4	12.9	2.6

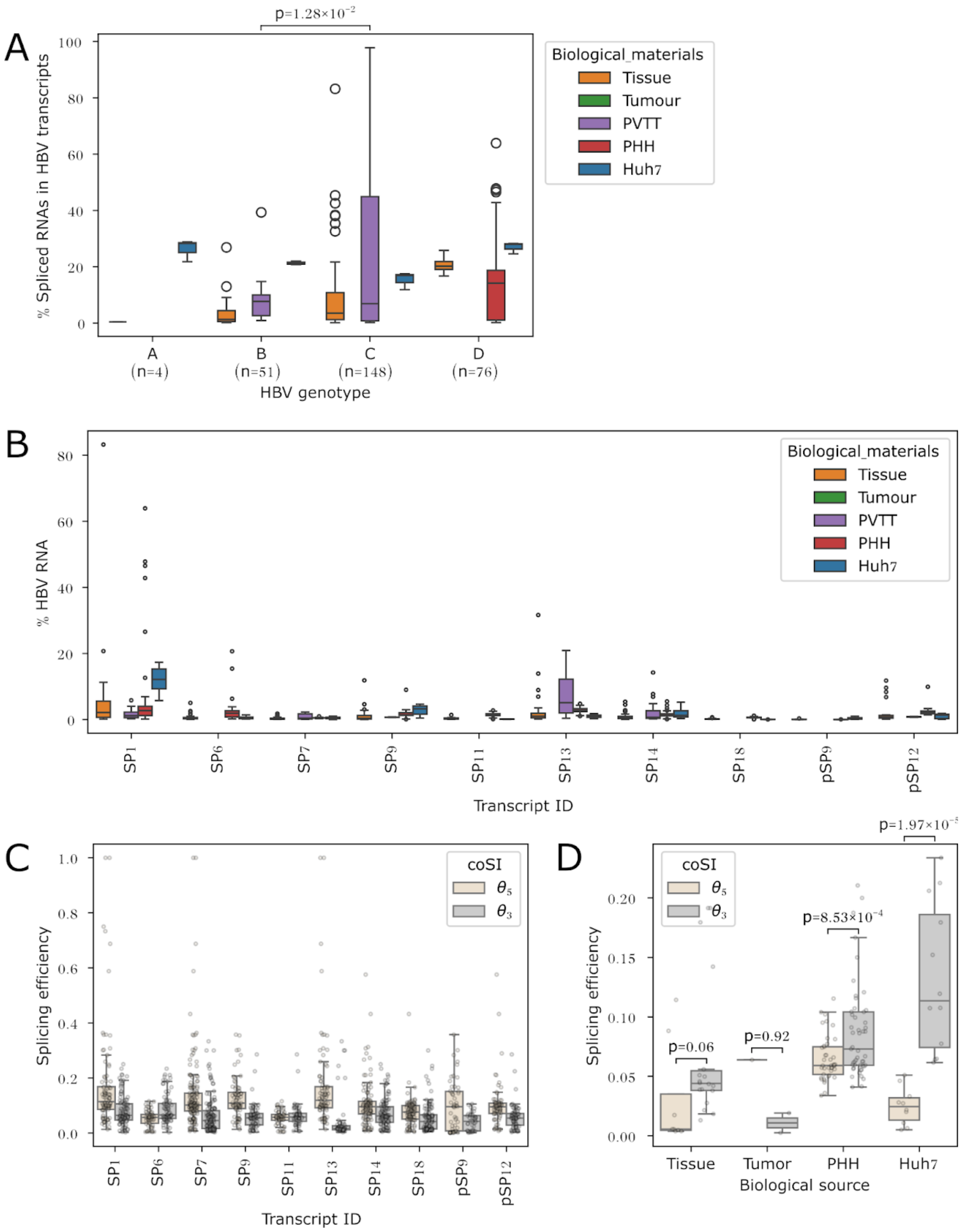

Fig S1. Splicing efficiency varies by transcript and genotype. (A) The levels of HBV splicing across genotypes and biological sources (279 transcriptomes). **(B)** Proportions of the 10 most common HBV splice variants detected in the transcriptomes. **(C)** HBV splice variants generally exhibit higher splicing efficiency at donor sites than acceptor sites, as indicated by higher completed splicing index (coSI) θ_5 scores compared to θ_3 scores, except for SP6. **(D)** SP6 shows higher θ_3 than θ_5 scores across samples. This pattern was less consistent in tumour samples with limited SP6 detection. P-value was derived from one-sided t-tests for independent samples.

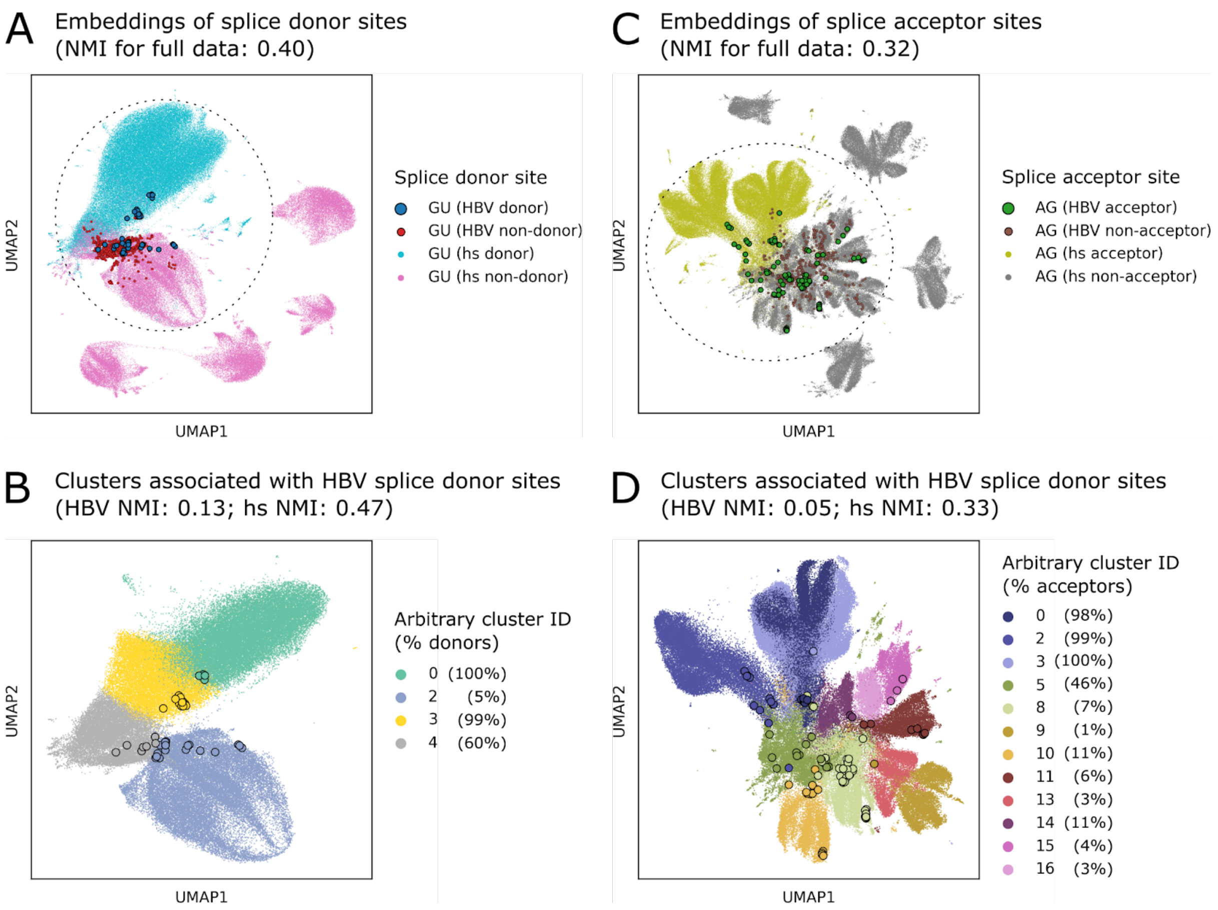

Fig S2. HBV splice sites remain distinct from host splice sites when using models fine-tuned on the full Spliceator dataset. Nucleotide embeddings for HBV and *H. sapiens* splice donor (**A, B**) and acceptor (**C, D**) sites were extracted from the final layer of SpliceBERT and processed using PCA, UMAP and the Leiden algorithm. Points representing HBV splice sites are outlined in black. Panels **B** and **D** show clusters marked by dotted circles in **A** and **C**, respectively. Normalised mutual information (NMI) scores in these panels indicate lower clustering consistency for HBV compared to host splice sites. This analysis complements Fig 2 by showing results from models fine-tuned on the full training set, which includes approximately 6% human splice sites.

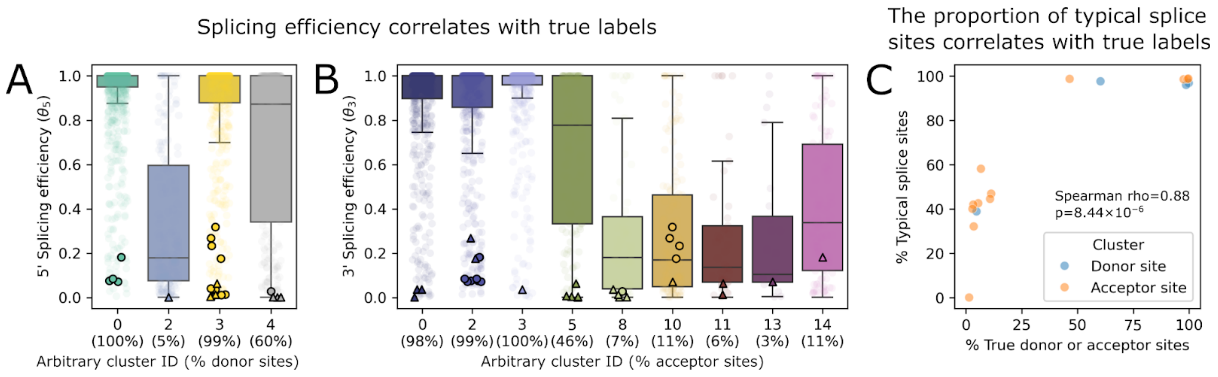

Fig S3. Splicing efficiency (A) and the proportion of typical splice sites (B) correlate with true label proportions in Leiden clusters. HBV splice sites are outlined in black, in which circles and triangles indicate well-known and novel HBV splice sites (**A** and **B**). Clusters were derived from the embedding space in Fig S2, where true labels within clusters include HBV and host splice sites. Percent typical host splice sites were calculated as the proportion of splice sites spanning the boundaries of exons and non-coding sequences (GU/AG dinucleotides) (**C**). This analysis complements Fig 3 by using models fine-tuned on the full Spliceator training set, which includes approximately 6% human splice sites.

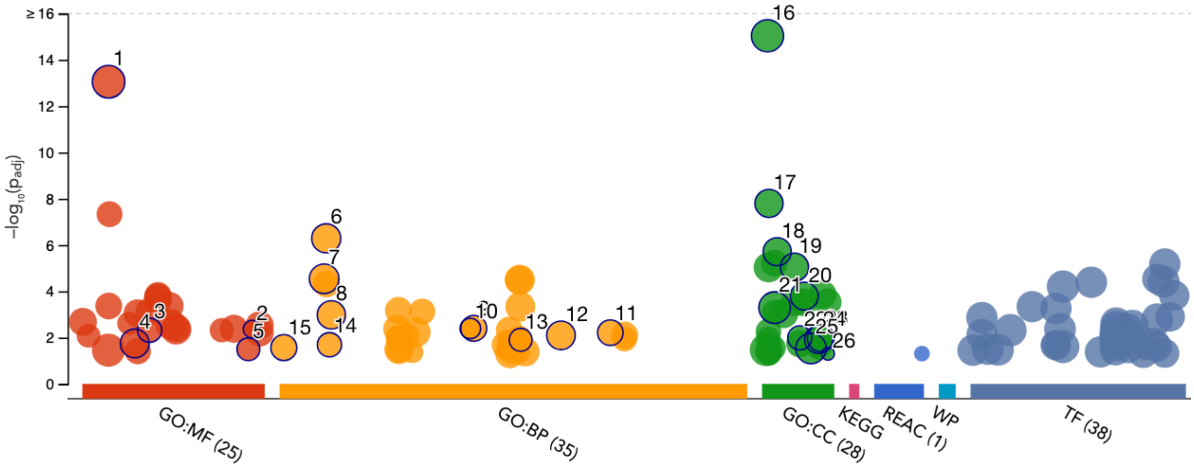

ID	Source	Term ID	Term Name	p_{adj} (query_1)
1	GO:MF	GO:0005515	protein binding	8.865×10^{-14}
2	GO:MF	GO:0140658	ATP-dependent chromatin remodeler activity	4.312×10^{-3}
3	GO:MF	GO:0030695	GTPase regulator activity	4.807×10^{-3}
4	GO:MF	GO:0016787	hydrolase activity	1.788×10^{-2}
5	GO:MF	GO:0120543	macromolecular conformation isomerase activity	3.185×10^{-2}
6	GO:BP	GO:0006996	organelle organization	5.165×10^{-7}
7	GO:BP	GO:0006810	transport	2.866×10^{-5}
8	GO:BP	GO:0007399	nervous system development	1.041×10^{-3}
9	GO:BP	GO:0044087	regulation of cellular component biogenesis	3.981×10^{-3}
10	GO:BP	GO:0043473	pigmentation	4.037×10^{-3}
11	GO:BP	GO:0097435	supramolecular fiber organization	6.094×10^{-3}
12	GO:BP	GO:0065008	regulation of biological quality	7.989×10^{-3}
13	GO:BP	GO:0051258	protein polymerization	1.240×10^{-2}
14	GO:BP	GO:0007264	small GTPase-mediated signal transduction	2.029×10^{-2}
15	GO:BP	GO:0000902	cell morphogenesis	2.716×10^{-2}
16	GO:CC	GO:0005737	cytoplasm	9.127×10^{-16}
17	GO:CC	GO:0005856	cytoskeleton	1.593×10^{-8}
18	GO:CC	GO:0030054	cell junction	1.934×10^{-6}
19	GO:CC	GO:0042995	cell projection	9.090×10^{-6}
20	GO:CC	GO:0070062	extracellular exosome	1.618×10^{-4}
21	GO:CC	GO:0016020	membrane	5.085×10^{-4}
22	GO:CC	GO:0045177	apical part of cell	1.032×10^{-2}
23	GO:CC	GO:0099080	supramolecular complex	1.047×10^{-2}
24	GO:CC	GO:0098588	bounding membrane of organelle	1.191×10^{-2}
25	GO:CC	GO:0071944	cell periphery	2.938×10^{-2}
26	GO:CC	GO:1904602	serotonin-activated cation-selective channel c...	4.999×10^{-2}

Fig S4. ***H. sapiens*** **genes containing CDS splice sites are significantly enriched for the Gene Ontology Biological Process term "organelle organization" (GO:0006996).**
